# Cross-national patterns of COVID-19 vaccination coverage by vaccine type

**DOI:** 10.1371/journal.pgph.0006682

**Published:** 2026-07-10

**Authors:** Walid Al-Zyoud, Dana Erekat, Mohammad Abudayah

**Affiliations:** 1 Department of Biomedical Engineering, School of Applied Medical Sciences, German Jordanian University, Amman, Jordan; 2 Department of Basic Sciences, School of Computing, German Jordanian University, Amman, Jordan; The University of Edinburgh, UNITED KINGDOM OF GREAT BRITAIN AND NORTHERN IRELAND

## Abstract

We examined whether COVID-19 vaccine type was associated with national full-vaccination coverage using 1034 country–vaccine entries from 195 countries derived from World Health Organization data. In a country-level analysis adjusting for World Bank income group, WHO region, and the number of vaccine products per country, neither mRNA vaccine availability (*p* = 0.051) nor the proportion of mRNA vaccines in a country’s portfolio (*p* = 0.164) was significantly associated with coverage. Vaccine-related variables added only one percentage point to the explained variance beyond structural factors ($\Delta R^{2}=0.011$). Unadjusted exploratory analyses showed that some vaccine products were more frequently recorded in high-coverage country contexts, but these patterns were substantially attenuated after adjustment for structural covariates. The apparent link between vaccine category and coverage largely reflected differences in economic capacity, geographic region, and deployment context rather than vaccine category itself.

## 1 Introduction

COVID-19 vaccination played a central role in the global response to the SARS-CoV-2 pandemic. Multiple vaccine platforms were developed and deployed in a relatively short period, including mRNA vaccines, viral-vector vaccines, inactivated vaccines, and protein-based vaccines. These platforms differ in their biological mechanisms, manufacturing pathways, storage requirements, and implementation demands. For example, mRNA vaccines induce an immune response by instructing host cells to produce the spike protein, whereas viral-vector vaccines use a modified virus to deliver the relevant genetic material. Protein-based vaccines, by contrast, rely on selected viral components rather than live or replicating material [1].

The global vaccination campaign was unprecedented in both scale and complexity. Although billions of doses were administered worldwide [2], vaccination coverage remained highly unequal across countries. Some countries achieved broad uptake within a relatively short period, whereas others experienced slower rollout and lower overall coverage. These differences have been associated with a wide range of structural factors, including vaccine supply, affordability, procurement power, delivery infrastructure, health-system capacity, and public confidence [3–7].

At the same time, vaccine types were not distributed uniformly across country settings. Some products were introduced more frequently in high-income countries, whereas others were more common in middle- or lower-income settings [8,9]. Moreover, vaccine types differed in several features that may have affected their deployment in practice, including storage conditions, dosing schedules, availability, and public perception. Clinical studies also reported varying efficacy profiles across products, particularly during the early phases of the pandemic [10–15]. For this reason, any observed relationship between vaccine type and national vaccination coverage is difficult to interpret directly.

In this study, the term “ecological” refers to analyses in which the unit of observation is a country or country–vaccine entry rather than an individual person. Accordingly, the associations reported here describe population-level patterns and should not be interpreted as individual-level effects.

In particular, a pooled ecological association between vaccine type and coverage may reflect not only vaccine-related characteristics, but also the broader structural conditions under which different vaccines were procured, distributed, and administered. Countries with stronger fiscal capacity and health-system infrastructure may both achieve higher coverage and preferentially deploy certain vaccine products. Conversely, countries facing logistical or supply constraints may rely on different vaccine portfolios while also experiencing lower coverage. It is therefore important to examine vaccine-type patterns cautiously and within their broader deployment context.

Motivated by this, the present study examines whether vaccine type was associated with differences in observed full-vaccination coverage in a global country-level dataset derived from World Health Organization data. We first explore the pooled pattern descriptively using one-way ANOVA, chi-square analysis with standardised residuals, and unadjusted logistic regression on an expanded country–vaccine-entry dataset. We then assess the robustness of these associations after adjustment for World Bank income group, WHO region, and the number of vaccine products per country, and after accounting for within-country clustering. As a primary confirmatory analysis, we collapse the dataset to the country level and examine whether vaccine-related variables explain meaningful variation in coverage beyond structural factors.

The objective of the study is not to infer an independent or causal effect of vaccine type on vaccination coverage. Rather, it is to describe whether vaccine-product presence was more commonly observed in higher- or lower-coverage country contexts, and to assess how far this population-level pattern persists after accounting for broad differences in national economic and regional context.

## 2 Methods

### 2.1 Data source

We analysed a curated global dataset derived from two publicly available World Health Organization (WHO) sources: the WHO COVID-19 vaccination uptake data and the WHO vaccine product introduction data [16]. The uptake dataset provided country-level information on full-vaccination coverage, whereas the product-introduction dataset identified the COVID-19 vaccine products recorded for use in each country. These two sources were linked by country code in order to construct the analytical dataset.

For the primary analysis, we used the latest available country-level vaccination coverage record from the WHO uptake data and merged it with the vaccine product information recorded for the same country. This yielded a country–vaccine-entry dataset in which each row represented a vaccine product recorded for a given country together with that country’s latest observed full-vaccination coverage value.

Because the unit of analysis was country-level rather than individual-level, all analyses were performed on aggregated national records. Accordingly, the objective was to examine broad comparative patterns in observed vaccination coverage across vaccine categories at the population level.

### 2.2 Data cleaning and aggregation

The WHO vaccination uptake data were used to obtain country-level full-vaccination coverage values, and the WHO vaccine product introduction data were used to identify the vaccine products recorded for each country. For the primary manuscript dataset, we retained the latest available vaccination coverage record for each country and merged it with the corresponding vaccine product records by country code.

The analytical dataset was therefore organised at the level of country–vaccine entries. Thus, when a country had recorded more than one vaccine product, the same latest country-level full-vaccination coverage value appeared in more than one row, once for each vaccine product associated with that country.

The primary analyses were performed on this latest-snapshot country–vaccine dataset. An expanded monthly country–vaccine panel was generated separately for reproducibility purposes, but it was not used as the main inferential dataset in the present study.

Duplicate country–vaccine product pairs were removed after harmonising country identifiers and vaccine-product labels. The resulting file contained 1090 country–vaccine entries. Of these, 9 entries had missing coverage values and were excluded, yielding an initial analytical sample of 1081 entries from 219 countries. A further 47 entries from 18 small territories lacking World Bank income classifications were excluded from the adjusted analyses, yielding a consistent analysis sample of 1034 entries from 195 countries. All results reported in this manuscript are based on this consistent sample unless otherwise noted.

### 2.3 Variable construction

The principal outcome considered in this study was full-vaccination coverage, measured as the percentage of the population reported by the WHO as fully vaccinated at the latest available country-level snapshot included in the analytical dataset.

For comparability across the chi-square and logistic regression analyses, coverage was also dichotomised into two groups: low coverage (0) and high coverage (1). The cutoff was defined using the median observed coverage in the final country–vaccine-entry dataset, thereby yielding a balanced binary outcome for comparative analysis.

This dichotomised formulation was used to distinguish vaccine categories more frequently associated with relatively lower or relatively higher observed coverage, and was not intended to represent a natural clinical or policy threshold.

Because dichotomisation may obscure variation in a continuous outcome [17], a supplementary sensitivity analysis was also performed using continuous full-vaccination coverage. In addition, World Bank income group [18] was incorporated in the adjusted analyses as a country-level structural variable in order to assess whether the observed vaccine-type patterns persisted after accounting for broad differences in national economic context.

Because WHO full-vaccination coverage reflects completion of the primary series as defined in each national setting, comparisons across vaccine categories should be interpreted cautiously, since the number of doses required for full vaccination could differ across vaccine products and countries.

### 2.4 ANOVA

A one-way analysis of variance (ANOVA) was applied as an exploratory comparison to examine whether mean full-vaccination coverage (continuous percentage) differed across vaccine types. Because the same country-level coverage value could appear more than once when a country had multiple vaccine products, this analysis was interpreted as a descriptive entry-level comparison rather than as a fully independent inferential test.

The analysis was conducted on the original continuous coverage values reported in the WHO dataset, using vaccine type as the grouping factor. The F-statistic was used to test for heterogeneity across vaccine groups, and eta squared ($\eta^{2}$) was reported as an effect-size measure indicating the proportion of total variation in continuous coverage explained by vaccine type. The corresponding mean plot with 95% confidence intervals provides a descriptive summary of how vaccine categories differed in their observed mean coverage levels.

### 2.5 Chi-square test and standardised residuals

To examine the association between vaccine type and the dichotomised coverage outcome, we constructed a contingency table of vaccine category by coverage group and applied the chi-square test of independence [19]. As with the ANOVA, this test was used descriptively because country–vaccine entries are not fully independent when several vaccine products are recorded for the same country. This provided a non-parametric assessment of whether the distribution of low-coverage and high-coverage observations differed across vaccine categories.

Standardised residuals were then calculated in order to identify which vaccine categories contributed most strongly to the overall chi-square association. Positive residuals indicate that a vaccine category appeared more often than expected in a given coverage group under the assumption of independence, whereas negative residuals indicate that it appeared less often than expected.

The residual heatmap was used as a visual summary of these deviations from expectation.

### 2.6 Unadjusted logistic regression

To complement the descriptive and contingency-based analyses, we fitted an unadjusted logistic regression model with coverage group as the binary outcome and vaccine type as the predictor. Vaccine categories with very small counts were combined into an “Other” group where necessary in order to reduce estimation instability and to avoid sparse-category effects in model fitting.

Dummy coding was used for the vaccine categories, and odds ratios (ORs) with 95% confidence intervals were calculated to summarise the direction and magnitude of the association between vaccine type and membership in the high-coverage group.

### 2.7 Adjusted logistic regression

The unadjusted logistic regression treated vaccine type as the sole predictor of membership in the high-coverage group. However, vaccine-platform deployment was closely associated with broader national conditions, particularly economic capacity and health-system resources, which may influence both vaccine availability and vaccination coverage. To examine whether the country-level association between vaccine type and coverage persisted after accounting for structural confounding, we fitted multivariable logistic regression models with increasing covariate sets.

The first adjusted model included vaccine type and World Bank income group (low, lower-middle, upper-middle, and high income). The second, fully adjusted model additionally included WHO region (AFRO, AMRO, EMRO, EURO, SEARO, WPRO) and the number of vaccine products introduced per country, a continuous variable serving as a proxy for procurement capacity and health-system reach. Adjusted odds ratios (aORs) with 95% confidence intervals were calculated for the vaccine categories.

In addition, we carried out a stratified analysis by fitting separate unadjusted logistic regression models within each income group in order to assess whether the vaccine-type–coverage association was similar across broad economic strata.

### 2.8 Continuous-outcome sensitivity analysis

Because vaccination coverage was dichotomised using a median split for the primary binary analyses, an additional sensitivity analysis was performed using continuous full-vaccination coverage as the outcome. Dichotomisation of a continuous variable may reduce information and obscure underlying variation [17]. Accordingly, we fitted an ordinary least-squares (OLS) regression model with continuous full-vaccination coverage as the dependent variable and vaccine type together with the full set of structural covariates (income group, WHO region, and portfolio size) as predictors.

The purpose of this analysis was to assess whether the general pattern remained similar when coverage was analysed on its original continuous scale.

### 2.9 Clustered standard errors

The analytical dataset was constructed at the level of country–vaccine entries. Consequently, countries that had recorded more than one vaccine product contributed more than one observation to the dataset. Since observations from the same country shared common structural characteristics, including health-system capacity, governance context, and economic conditions, the usual independence assumption for regression errors may be violated.

We therefore examined cluster-robust standard errors grouped by country [20]. However, because the outcome (national coverage) has zero within-country variation, the cluster-robust variance estimator produced unreliable results for vaccine-type predictors (see Sect 4.5). The country-level analysis described below was therefore adopted as the primary approach for addressing within-country dependence.

### 2.10 Country-level analysis (primary specification)

Because the analytical dataset contained repeated entries for the same country, the effective number of independent units was substantially smaller than the total number of rows. The entry-level analyses described above are therefore presented as exploratory comparisons. As the primary confirmatory specification, we collapsed the dataset to one row per country and fitted an OLS regression with continuous full-vaccination coverage as the outcome. In this specification, the vaccine-type categorical predictors were replaced by the proportion of mRNA vaccines in each country’s portfolio and a binary indicator for mRNA availability. The structural covariates (income group, WHO region, and portfolio size) were retained. This analysis provided a direct test of whether vaccine platform explained meaningful variation in coverage beyond structural factors, without the repeated-entry dependence or the dichotomisation instability that affected the entry-level logistic models.

### 2.11 Conceptual allocation perspective

In addition to the empirical analyses, we included a conceptual allocation perspective to illustrate how unequal distribution of vaccine platforms across countries with different economic and delivery capacities may contribute to the observed coverage patterns. In particular, vaccine products were not introduced uniformly across national settings, and some platforms were more frequently recorded in countries with greater fiscal, logistical, and health-system resources.

**Fig 1:**
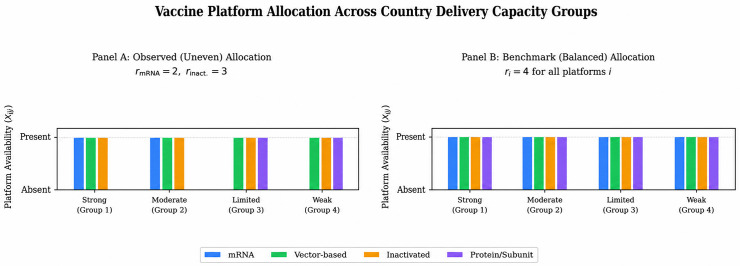
Conceptual illustration of vaccine platform allocation across country delivery capacity groups. Panel A depicts the observed uneven allocation, in which mRNA platforms were concentrated in stronger-capacity settings. Panel B depicts a hypothetical balanced allocation in which all platforms are available in all groups.

This framework was interpretive only and was not used as part of the statistical estimation. Its role was to provide a conceptual perspective through which the empirical findings may be understood (Fig 1).

### 2.12 Software

All statistical analyses were conducted in Python using pandas, NumPy, SciPy, and statsmodels. Visualisations were created with matplotlib. The dataset-construction and analysis workflow was implemented in a reproducible computational environment, and the cleaned analytical dataset together with the data-construction script may be provided as supplementary materials.

## 3 Results

The consistent analysis sample consisted of 1034 country–vaccine entries from 195 countries (see Sect 2.2 for the sample-reduction chain). Because this dataset contains repeated entries for countries with multiple vaccine products, the analyses in this section are presented as exploratory descriptive comparisons only. Their *p*-values should therefore be interpreted cautiously. The confirmatory country-level analysis, which uses one observation per country, is reported in Sect 4.

### 3.1 ANOVA

ANOVA revealed statistically significant heterogeneity in continuous full-vaccination coverage across vaccine types (*F*(17,1016)=3.59, $p=1.17\times 10^{-6}$, $\eta^{2}=0.057$), indicating that vaccine type explained approximately 5.7% of the total variation in vaccination coverage values (Tables 1 and 2). Because countries with multiple vaccine products contributed repeated observations, this *p*-value should be interpreted descriptively rather than as a fully independent inferential test.

**Table 1:** ANOVA Test — Impact of Vaccine Type on Vaccination Coverage.

Source	Sum of Squares	df	F-value	p-value	Interpretation
Vaccine type	31398.84	17	3.59	$1.17\times 10^{-6}$	Statistically significant heterogeneity across vaccine types
Residual	522580.06	1016	–	–	Remaining unexplained variation

**Table 2:** Effect Size (Eta Squared) for ANOVA.

Source	Sum Sq	df	F	p-value	$\eta^{2}$
Vaccine type	31398.84	17	3.59	$1.17\times 10^{-6}$	0.0567
Residual	522580.06	1016	–	–	0.9433

**Fig 2:**
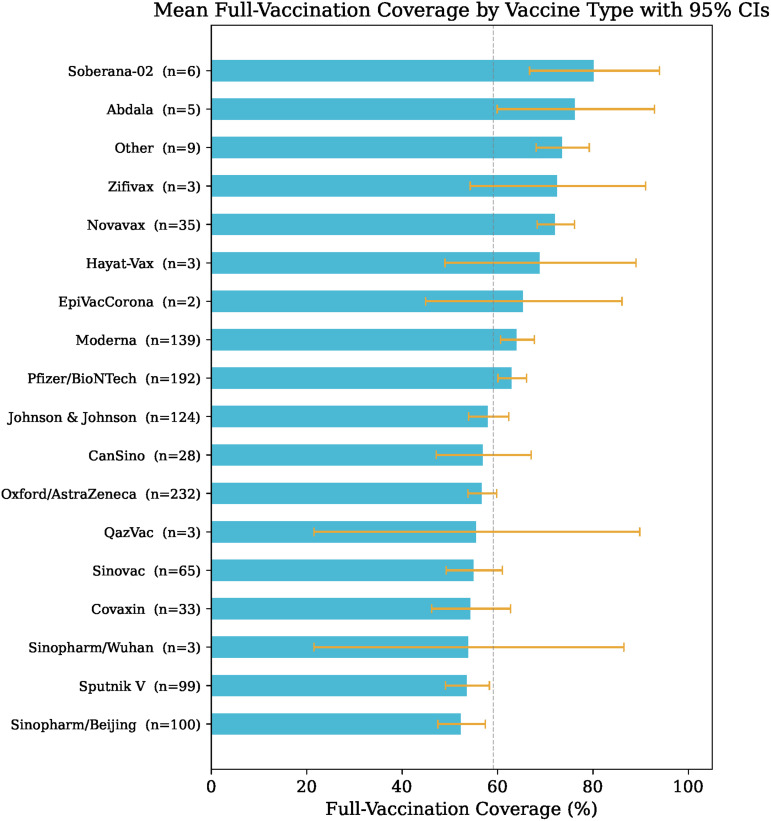
Mean full-vaccination coverage (continuous percentage) by vaccine type with 95% confidence intervals. The vertical dashed line indicates the overall mean coverage. Sample sizes for each vaccine category are shown in parentheses.

Thus, although the ANOVA detected statistically significant differences across vaccine types, the corresponding effect size remained limited. This suggests that vaccine type captured only part of the observed variation in the dataset. Fig 2 presents the mean full-vaccination coverage by vaccine type together with 95% confidence intervals.

### 3.2 Chi-square test and standardised residuals

Using the harmonised vaccine-family categories, the chi-square test showed a statistically significant association between vaccine type and coverage group, with $\chi^{2}(17)=53.80$ and $p=1.07\times 10^{-5}$. This result should also be interpreted descriptively, since the contingency table is based on country–vaccine entries rather than independent country-level observations. Standardised residuals were then used to identify which vaccine categories contributed most strongly to this association.

Novavax (+2.81), Moderna (+1.93), and Pfizer/BioNTech (+1.26) were overrepresented among entries from high-coverage country contexts, whereas Sinopharm/Beijing (-1.61), Sputnik V (-1.54), and Oxford/AstraZeneca (-1.41) were more frequent among entries from low-coverage country contexts (Table 3). Categories with very small counts were interpreted cautiously.

**Table 3:** Chi-Square Test — Association Between Vaccine Type and Coverage Group (*N* = 1034).

Vaccine	Low (0)	High (1)	Res. (Low)	Res. (High)	Interpretation
Pfizer/BioNTech	75	102	−1.28	+1.26	More frequent in high-coverage contexts
Moderna	49	83	−1.97	+1.93	More frequent in high-coverage contexts
Johnson & Johnson	56	60	−0.13	+0.13	Neutral
Oxford/AstraZeneca	124	98	+1.43	−1.41	More frequent in low-coverage contexts
Sinopharm/Beijing	59	38	+1.64	−1.61	More frequent in low-coverage contexts
Sputnik V	59	39	+1.56	−1.54	More frequent in low-coverage contexts
Sinovac	37	26	+1.09	−1.07	Mild tendency to lower coverage
Covaxin	19	14	+0.69	−0.68	Mild tendency to lower coverage
Novavax	5	29	−2.86	+2.81	More frequent in high-coverage contexts
CanSino	15	13	+0.34	−0.33	Neutral
Abdala	2	3	−0.29	+0.29	Neutral; very small sample
Soberana-02	1	5	−1.13	+1.11	Small sample
Sinopharm/Wuhan	2	1	+0.43	−0.43	Neutral; tiny sample
Hayat-Vax	1	2	−0.39	+0.38	Neutral; tiny sample
QazVac	1	2	−0.39	+0.38	Neutral; tiny sample
Zifivax	1	2	−0.39	+0.38	Neutral; tiny sample
EpiVacCorona	1	1	+0.02	−0.02	Neutral; tiny sample
Other	1	8	−1.63	+1.60	Mixed sparse group

**Fig 3:**
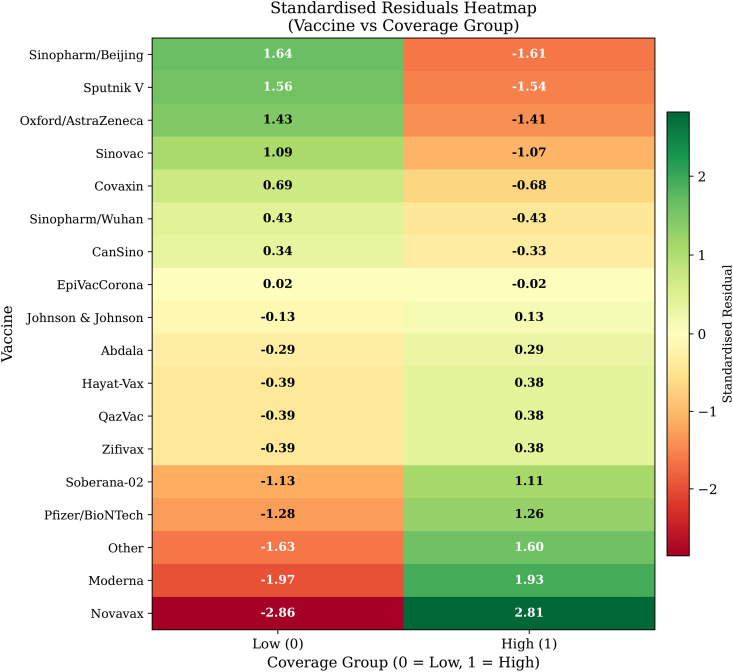
Standardised residuals heatmap for the vaccine type by coverage group contingency table. Green cells indicate overrepresentation and red cells indicate underrepresentation relative to the expected frequency under independence.

The residual heatmap provides a visual representation of the same pattern. Positive residuals indicate vaccine categories that are more common than expected in a given coverage group, whereas negative residuals indicate categories that are less common than expected under independence (Fig 3).

**Fig 4:**
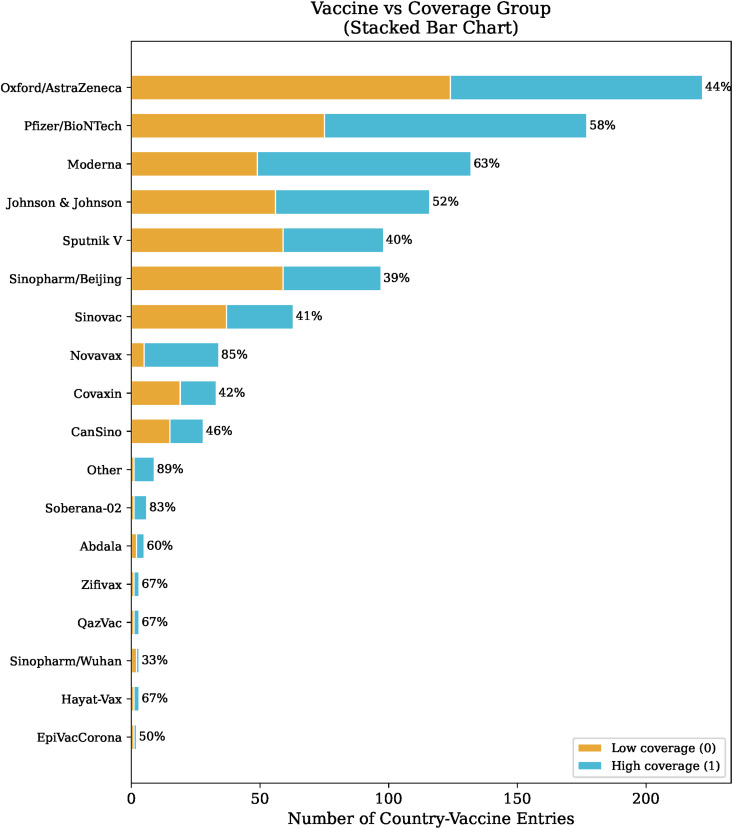
Stacked bar chart showing the number of country–vaccine entries in the low-coverage and high-coverage groups by vaccine category. Percentages indicate the proportion of entries in the high-coverage group.

The stacked bar chart confirms the same descriptive tendency, with Pfizer/BioNTech, Moderna, and Novavax more frequently represented among high-coverage observations, whereas Oxford/AstraZeneca, Sinopharm/Beijing, and Sputnik V were more concentrated in the low-coverage group (Fig 4).

### 3.3 Unadjusted logistic regression

To complement the descriptive and contingency-based analyses, we fitted an unadjusted logistic regression model with the binary coverage group as the outcome. Vaccine categories with fewer than 10 observations were collapsed into a single “Other” category to ensure model stability; Novavax (*n* = 34) was retained as a separate category. Because the binary outcome is repeated within countries, these odds ratios should be interpreted as exploratory entry-level contrasts rather than as product-specific effects. The results showed that entries for Moderna had higher odds of belonging to the high-coverage group (OR=2.14, p=0.001), as were Pfizer/BioNTech (OR=1.72, p=0.008) and Novavax (OR=7.34, p<0.001). The collapsed “Other” category also showed higher odds relative to the reference category (OR=3.04, p=0.005).

By contrast, Johnson & Johnson (OR=1.36, p=0.185), Sinopharm/Beijing (OR=0.81, p=0.409), Sinovac (OR=0.89, p=0.685), Sputnik V (OR=0.84, p=0.469), and Covaxin (OR=0.93, p=0.853) did not differ significantly from the reference category (Table 4).

**Table 4:** Unadjusted Logistic Regression Results (*N* = 1034; reference: Oxford/AstraZeneca; rare vaccines collapsed into “Other”).

Vaccine	OR	95% CI	p-value	Interpretation
Oxford/AstraZeneca	*(reference)*			–
Moderna	2.14	[1.38–3.33]	0.001	More frequent in high-coverage contexts
Pfizer/BioNTech	1.72	[1.15–2.56]	0.008	More frequent in high-coverage contexts
Novavax	7.34	[2.74–19.66]	<0.001	More frequent in high-coverage contexts
Johnson & Johnson	1.36	[0.86–2.13]	0.185	NS
Sinopharm/Beijing	0.81	[0.50–1.33]	0.409	NS
Sinovac	0.89	[0.50–1.57]	0.685	NS
Sputnik V	0.84	[0.52–1.36]	0.469	NS
Covaxin	0.93	[0.45–1.95]	0.853	NS
CanSino	1.10	[0.50–2.41]	0.819	NS
Other (collapsed)	3.04	[1.39–6.65]	0.005	More frequent in high-coverage contexts

**Fig 5:**
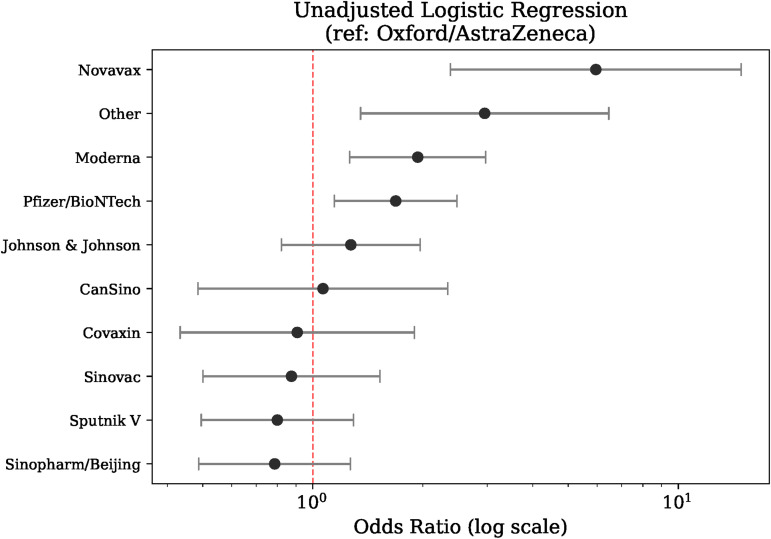
Forest plot of unadjusted odds ratios for membership in the high-coverage group by vaccine category, with Oxford/AstraZeneca as the reference. The vertical dashed line at 1 indicates no association. Error bars represent 95% confidence intervals.

The corresponding forest plot summarises these estimated odds ratios visually (Fig 5).

### 3.4 Master summary table

Table 5 summarises the findings across the three primary analytical approaches. Taken together, these pooled analyses showed that vaccine type was associated with observed differences in vaccination coverage, although the ANOVA effect size was modest ($\eta^{2}=0.057$).

**Table 5:** Master summary table.

Method	Statistic/Effect	p-value	Key Result	Interpretation
ANOVA	$F(17,1016)=3.59,\;\eta^{2}=0.057$	$1.17\times 10^{-6}$	Exploratory entry-level differences were observed across vaccine types	vaccine-product presence was associated with the national coverage context in which it was recorded, but the effect size was modest
Chi-Square	$\chi^{2}(17)=53.80$	$1.07\times 10^{-5}$	An exploratory entry-level association was observed between vaccine type and coverage group	Some vaccine categories were more common in high-coverage observations, whereas others were more common in low-coverage observations
Standardised residuals	Novavax (+2.81), Moderna (+1.93), Pfizer/BioNTech (+1.26), Sinopharm/Beijing (−1.61), Sputnik V (−1.54), Oxford/AstraZeneca (−1.41)	–	mRNA vaccines and Novavax were more represented among entries from high-coverage country contexts, whereas several other categories were more represented in the low-coverage group	Identifies the categories contributing most strongly to the chi-square association
Unadjusted logistic regression	Moderna OR 2.14, Pfizer/BioNTech OR 1.72, Novavax OR 7.34	See Table 4	Several vaccine categories differed in their odds of membership in the high-coverage group, although only a subset reached statistical significance	Quantifies the unadjusted association; these estimates do not account for structural confounders

## 4 Adjusted entry-level analyses and primary country-level analysis

This section reports adjusted analyses undertaken to assess whether the exploratory associations identified above persisted after accounting for structural covariates. The entry-level models (Sects 4.1–4.5) progressively adjust the country–vaccine-entry dataset. The country-level analysis (Sect 4.6), which eliminates the repeated-entry structure, serves as the primary confirmatory specification.

### 4.1 Income-adjusted logistic regression

Table 6 presents the multivariable logistic regression including both vaccine type and World Bank income group. Relative to the unadjusted model in Table 4, the vaccine-type associations were substantially attenuated after income adjustment.

**Table 6:** Income-Adjusted Logistic Regression Results (*N* = 1034; reference: Oxford/AstraZeneca for vaccine type; high income for income group).

Predictor	aOR	95% CI	p-value	Sig.
**Vaccine type**				
Moderna	1.53	[0.94–2.49]	0.088	
Pfizer/BioNTech	1.21	[0.78–1.88]	0.403	
Johnson & Johnson	1.11	[0.67–1.83]	0.685	
Sinopharm/Beijing	1.10	[0.65–1.86]	0.723	
Sinovac	1.13	[0.61–2.08]	0.699	
Sputnik V	1.01	[0.60–1.71]	0.963	
Covaxin	1.19	[0.53–2.67]	0.682	
Novavax	2.79	[0.99–7.87]	0.052	
Other (collapsed)	4.12	[1.83–9.24]	0.001	***
**Income group**				
Upper-middle income	0.18	[0.13–0.26]	<0.001	***
Lower-middle income	0.20	[0.14–0.29]	<0.001	***
Low income	0.05	[0.02–0.09]	<0.001	***

After adjustment for income group, the odds ratios for the major vaccine categories moved substantially toward the null and none of the individually listed vaccine-type coefficients remained statistically significant. By contrast, income group itself remained strongly associated with coverage, with upper-middle-, lower-middle-, and low-income settings showing markedly lower odds of membership in the high-coverage group relative to high-income settings.

**Fig 6:**
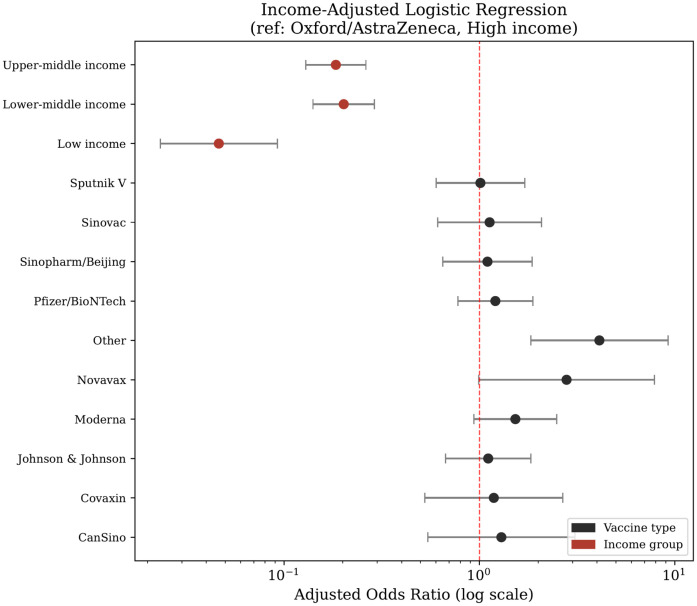
Forest plot of income-adjusted odds ratios for membership in the high-coverage group. Vaccine-type coefficients (black) use Oxford/AstraZeneca as reference; income-group coefficients (red) use high income as reference. The vertical dashed line at 1 indicates no association.

The increase in pseudo-*R*^2^ from the unadjusted model to the adjusted model further suggested that national income explained substantially more variation in coverage than vaccine type alone. Overall, these findings support the interpretation that the pooled unadjusted associations were influenced strongly by broader structural differences across country settings rather than by vaccine category considered in isolation. The corresponding income-adjusted odds ratios are displayed in Fig 6.

### 4.2 Fully adjusted logistic regression

To assess whether the income-adjusted findings were robust to the inclusion of additional structural covariates, we fitted a fully adjusted logistic regression model including vaccine type, World Bank income group, WHO region, and the number of vaccine products per country (Table 7).

**Table 7:** Fully Adjusted Logistic Regression Results (reference: Oxford/AstraZeneca, high income, EURO).

Predictor	aOR	95% CI	p-value	Sig.
**Vaccine type**				
Moderna	1.58	[0.89–2.82]	0.118	
Pfizer/BioNTech	1.33	[0.79–2.24]	0.276	
Johnson & Johnson	1.14	[0.63–2.05]	0.674	
Sinopharm/Beijing	1.04	[0.53–2.01]	0.919	
Sinovac	1.22	[0.57–2.60]	0.606	
Sputnik V	1.28	[0.67–2.45]	0.446	
Covaxin	1.50	[0.56–4.02]	0.425	
Novavax	1.82	[0.62–5.28]	0.274	
CanSino	1.80	[0.65–5.03]	0.259	
Other (collapsed)	6.24	[2.43–16.03]	<0.001	***
**Income group**				
Upper-middle income	0.10	[0.06–0.15]	<0.001	***
Lower-middle income	0.04	[0.02–0.07]	<0.001	***
Low income	0.03	[0.02–0.08]	<0.001	***
**WHO region**				
AFRO	2.58	[1.26–5.27]	0.009	**
AMRO	1.36	[0.86–2.17]	0.192	
EMRO	0.58	[0.32–1.07]	0.084	
SEARO	99.84	[31.57–315.69]	<0.001	***
WPRO	49.90	[22.01–113.14]	<0.001	***
**Other**				
No. of vaccine products	1.24	[1.14–1.36]	<0.001	***

The fully adjusted model showed substantially improved fit compared with the income-only model (pseudo-*R*^2^ from 0.153 to 0.345; AIC from 1242 to 979). None of the individually listed vaccine-type coefficients reached statistical significance, whereas income group, WHO region, and portfolio size were all strongly associated with coverage. Countries in the SEARO and WPRO regions showed markedly higher odds of membership in the high-coverage group, and each additional vaccine product was associated with 24% higher odds of high coverage. However, the very large adjusted odds ratios for SEARO and WPRO (approximately 100 and 50, respectively) suggest possible instability in the dichotomised logistic specification due to sparse cells or near-separation in these regional strata. The continuous-outcome model reported below does not exhibit this behaviour and should be regarded as the more stable specification for interpreting regional effects.

### 4.3 Stratified analysis by income group

Table 8 presents the unadjusted logistic regression results within each World Bank income stratum for selected vaccine categories, using Oxford/AstraZeneca as the reference category. Within each stratum, the direction and magnitude of the odds ratios varied, and none of the selected vaccine categories showed a consistent pattern of association across income groups. Estimates in the lower-middle and low income strata should be interpreted with particular caution due to sparse vaccine-category counts.

**Table 8:** Unadjusted Logistic Regression by Income Group (selected vaccine types, reference: Oxford/AstraZeneca).

Income group	Vaccine	OR	p-value
High income (*n* = 262)	Moderna	1.60	0.229
	Pfizer/BioNTech	1.49	0.268
	Sinovac	1.71	0.517
	Sputnik V	1.39	0.598
Upper-middle income (*n* = 188)	Moderna	1.46	0.419
	Pfizer/BioNTech	1.01	0.988
	Sinovac	1.10	0.853
	Sputnik V	0.62	0.353
Lower-middle income (*n* = 201)	Moderna	1.37	0.465
	Pfizer/BioNTech	1.11	0.803
	Sinovac	1.03	0.961
	Sputnik V	1.17	0.693
Low income (*n* = 64)	Moderna	1.44	0.767
	Pfizer/BioNTech	1.24	0.862
	Sinovac	1.24	0.862
	Sputnik V	0.79	0.844

Overall, the lack of a stable within-stratum pattern supports the interpretation that the pooled unadjusted associations were influenced strongly by the unequal distribution of vaccine categories across countries with different economic capacities. Once the comparison was restricted within broad income strata, the apparent vaccine-type differences became less consistent and were generally attenuated.

### 4.4 Continuous-outcome sensitivity analysis

Table 9 presents the fully adjusted OLS regression results using continuous full-vaccination coverage as the outcome.

**Table 9:** OLS Regression with Continuous Coverage as Outcome (fully adjusted; reference: Oxford/AstraZeneca, high income, EURO).

Predictor	Coef.	95% CI	p-value
**Vaccine type**			
Moderna	+1.34	[−2.35, + 5.03]	0.475
Pfizer/BioNTech	+0.68	[−2.69, + 4.06]	0.690
Johnson & Johnson	−1.13	[−4.91, + 2.66]	0.559
Sinopharm/Beijing	+0.39	[−3.65, + 4.42]	0.851
Sinovac	+2.36	[−2.41, + 7.13]	0.332
Sputnik V	+1.90	[−2.21, + 6.02]	0.364
Covaxin	+4.03	[−2.29, + 10.34]	0.211
Novavax	−1.85	[−8.11, + 4.42]	0.563
CanSino	+5.03	[−1.80, + 11.85]	0.149
Other (collapsed)	+14.56	[+8.37, +20.74]	<0.001
**Income group**			
Upper-middle income	−25.67	[−28.51, −22.84]	<0.001
Lower-middle income	−29.12	[−32.30, −25.95]	<0.001
Low income	−32.55	[−37.16, −27.93]	<0.001
**WHO region**			
AMRO	+9.48	[+6.22, +12.75]	<0.001
SEARO	+29.63	[+24.70, +34.55]	<0.001
WPRO	+25.82	[+22.01, +29.62]	<0.001
EMRO	−10.09	[−14.02, −6.16]	<0.001
AFRO	+2.47	[−2.06, + 7.00]	0.285
No. of vaccine products	+2.16	[+1.60, +2.72]	<0.001

The continuous-outcome analysis was broadly consistent with the fully adjusted logistic regression. After accounting for structural covariates, the major vaccine-type coefficients were small and not statistically significant, whereas income group and WHO region remained strongly associated with coverage. Model fit improved substantially relative to the income-only specification (*R*^2^ = 0.488, adjusted *R*^2^ = 0.479, compared with *R*^2^ = 0.290 for the income-only model).

### 4.5 Note on clustered standard errors

We initially re-estimated the fully adjusted logistic regression using cluster-robust standard errors grouped by country [20]. However, inspection of the results revealed that the clustered standard errors for the vaccine-type predictors were *smaller* than the corresponding naive standard errors, producing misleadingly narrow confidence intervals and spurious significance. This anomaly arises because the outcome (national coverage) has zero within-country variation — each country contributes the same coverage value to every vaccine row — so the naive model overestimates the residual variance for within-country predictors. Clustering then “corrects” this overestimate, but the resulting inference is unreliable: the narrower standard errors do not reflect genuine precision but rather a breakdown of the variance decomposition when the outcome is constant within clusters. For the structural covariates (income group, WHO region, portfolio size), which vary only between countries, the clustered standard errors behaved as expected (wider than naive). Because the cluster-robust estimator is not appropriate for evaluating vaccine-type associations in this data structure, we rely instead on the country-level analysis reported below as the primary confirmatory specification.

### 4.6 Country-level analysis (primary specification)

To address the repeated-entry structure directly, this primary specification collapsed the dataset to one row per country (*N* = 195). The vaccine-type categorical predictor was therefore replaced with two country-level portfolio variables: the proportion of mRNA vaccines in each country’s vaccine portfolio and a binary indicator of mRNA availability (Table 10).

**Table 10:** Country-Level OLS Regression (*N* = 195 countries; reference: high income, EURO).

Predictor	Coef.	95% CI	p-value
Has mRNA (binary)	−12.52	[−25.07, + 0.03]	0.051
Proportion mRNA	+16.41	[−6.78, + 39.60]	0.164
No. of vaccine products	+2.32	[+0.81, +3.83]	0.003
**Income group**			
Upper-middle income	−19.60	[−26.81, −12.38]	<0.001
Lower-middle income	−27.32	[−35.90, −18.74]	<0.001
Low income	−27.27	[−39.02, −15.52]	<0.001
**WHO region**			
SEARO	+29.42	[+16.28, +42.56]	<0.001
WPRO	+23.95	[+15.07, +32.84]	<0.001

At the country level, neither mRNA availability (*p* = 0.051) nor the proportion of mRNA vaccines in a country’s portfolio (*p* = 0.164) was significantly associated with coverage after adjustment for structural covariates. Adding vaccine-related variables to a structural-only model increased *R*^2^ by only 1.1 percentage points (from 0.478 to 0.488), confirming that vaccine platform explained very little variation beyond what was accounted for by income group, WHO region, and portfolio size. The cross-tabulation of mRNA availability by income group further illustrated the confounding: 98.5% of high-income countries had access to mRNA vaccines, compared with only 28.6% of low-income countries. Because this specification uses one observation per country and a continuous outcome, it avoids both the repeated-entry dependence and the dichotomisation instability that affected the entry-level logistic models, and should be regarded as the most reliable of the analyses presented.

### 4.7 Summary

Table 11 summarises the principal vaccine-type estimates across model specifications. The robustness analyses indicate that the pooled population-level association between vaccine type and coverage was substantially influenced by structural covariates and by the repeated country structure of the dataset. Vaccine categories that appeared positively associated with membership in the high-coverage group in the unadjusted pooled analysis were attenuated after adjustment, and most vaccine-type coefficients were no longer statistically significant in the fully adjusted model.

**Table 11:** Comparison of Key Vaccine-Type Estimates Across Model Specifications (*N* = 1034).

Vaccine	Unadj. OR	Inc.-adj. aOR	Full-adj. aOR	Full OLS coef.
Moderna	2.14***	1.53	1.58	+1.34
Pfizer/BioNTech	1.72**	1.21	1.33	+0.68
Novavax	7.34***	2.79	1.82	−1.85
Sinovac	0.89	1.13	1.22	+2.36
Sputnik V	0.84	1.01	1.28	+1.90
Covaxin	0.93	1.19	1.50	+4.03
Sinopharm/Beijing	0.81	1.10	1.04	+0.39
Other (collapsed)	3.04**	4.12***	6.24***	$+14.56^{***}$

^**^p < 0.01, ^***^p < 0.001. Full-adj. = adjusted for income group, WHO region, and portfolio size. OLS coefficients denote change in continuous coverage (percentage points) relative to Oxford/AstraZeneca. All models fitted on the consistent analysis sample (*N* = 1034 entries from 195 countries).

By contrast, income group, WHO region, and portfolio size remained strong predictors across model specifications, together explaining nearly half the variation in observed coverage (*R*^2^ = 0.488 in the fully adjusted OLS). The country-level analysis confirmed that vaccine-related variables added only marginal explanatory power beyond structural factors.

## 5 Discussion

The analyses presented in this study examined whether vaccine-product presence was associated with the national coverage contexts in which products were recorded. In a country–vaccine-entry dataset, the pooled unadjusted analyses showed statistically significant heterogeneity across vaccine categories, with mRNA vaccines (Moderna and Pfizer/BioNTech) and Novavax appearing more frequently in high-coverage country contexts, whereas Oxford/AstraZeneca, Sinopharm/Beijing, and Sputnik V were more frequent in low-coverage country contexts. However, the ANOVA effect size was modest ($\eta^{2}=0.057$), and the robustness analyses consistently showed that these patterns were substantially attenuated after adjustment for structural covariates.

### 5.1 Interpretation

In this sense, vaccine type appeared primarily as a marker of deployment context rather than as an independent determinant of national coverage. In the primary country-level analysis (*N* = 195), which eliminated the repeated-entry structure and used continuous coverage as the outcome, neither mRNA vaccine availability nor the proportion of mRNA vaccines in a country’s portfolio was significantly associated with coverage after adjustment for income group, WHO region, and portfolio size. Vaccine-related variables added only approximately one percentage point to the explained variance beyond structural factors alone (*R*^2^ from 0.478 to 0.488). The entry-level exploratory analyses supported this conclusion: in the fully adjusted logistic model, none of the individually listed vaccine-type coefficients reached statistical significance, and model fit improved substantially when structural covariates were added (pseudo-*R*^2^ from 0.153 to 0.345).

By contrast, income group remained strongly associated with coverage across all model specifications, with low-income countries showing markedly lower odds of membership in the high-coverage group relative to high-income settings. WHO region contributed additional explanatory power, with countries in the South-East Asia and Western Pacific regions showing substantially higher coverage after adjusting for income. The number of vaccine products introduced per country — a proxy for procurement capacity and health-system reach — was likewise positively associated with coverage. Together, these structural variables explained nearly half of the observed variation in full-vaccination coverage.

These patterns are consistent with the broader literature on global vaccination inequity. Wouters et al. [3] documented that vaccine access was shaped primarily by production capacity, affordability, and procurement power, while Lazarus et al. [4] and Sallam [5] highlighted the additional role of public confidence and acceptance. The present findings complement these studies by showing that, at the country level, the apparent association between vaccine type and coverage was largely a reflection of the structural conditions under which different vaccine categories were deployed. In particular, mRNA vaccines were available in 98.5% of high-income countries but in only 28.6% of low-income countries, creating a near-complete confound between vaccine platform and national economic capacity.

These findings also help contextualise debates about vaccine hesitancy, misinformation, and public scrutiny of different vaccine platforms. They should not be read as showing that public attitudes were unimportant. Rather, in this cross-national analysis, vaccine-platform variables did not explain substantial additional variation in coverage after adjustment for income group, WHO region, and portfolio size. Public confidence, hesitancy, and platform-specific perceptions may still have influenced uptake within countries and over time, but their effects were likely embedded within broader structural and deployment contexts that shaped which vaccines were available, trusted, and delivered.

The persistent significance of the collapsed “Other” category across all model specifications warrants specific comment. This category comprised a heterogeneous set of sparse vaccine entries, including Cuban vaccines (Abdala, Soberana-02), Chinese domestic vaccines (Zifivax, Sinopharm/Wuhan), Central Asian products (QazVac, EpiVacCorona), and several unharmonised product labels. The countries contributing to this group included Cuba (89% coverage), China (87%), Nicaragua (92%), and Vietnam (88%), many of which were vaccine-manufacturing countries that both developed and deployed their own products domestically. These countries tended to achieve high coverage for structural reasons — including strong centralised public health systems and domestic production capacity — that are not fully captured by income group, WHO region, or portfolio size. The persistent coefficient therefore most likely reflects this self-manufacturing structural advantage and the heterogeneous composition of the collapsed category, rather than any intrinsic property of the vaccines themselves. This interpretation is supported by the observation that no individually listed vaccine category retained significance in the fully adjusted models.

### 5.2 Limitations

Several limitations should be acknowledged. First, the study was ecological in nature in the sense defined above: all observations were aggregated at the country or country–vaccine-entry level, and the results should not be interpreted as evidence of individual-level effects of vaccine type on vaccination behaviour. The ecological associations reported here may be subject to aggregation bias and ecological fallacy, and should be understood as descriptive patterns rather than causal estimates.

Second, the exploratory analyses were conducted on a country–vaccine-entry dataset in which countries with multiple vaccine products contributed more than one observation. The same national coverage value was repeated across products, meaning the exposure (vaccine type) was only loosely linked to the outcome (national coverage). Therefore, entry-level estimates should not be interpreted as measuring the uptake or effectiveness of individual vaccine products. The primary country-level analysis addressed this by collapsing to one row per country, but the ANOVA and chi-square results reported in Sect 3 did not account for within-country clustering, and their p-values should be interpreted conservatively.

Third, the set of structural covariates, while broader than income group alone, remained limited. Factors such as health expenditure per capita, COVAX participation, cold-chain infrastructure, and vaccine hesitancy indices were not included, and residual confounding cannot be ruled out. The adjusted results should therefore be interpreted as partial country-level adjustments rather than fully confounder-controlled estimates.

Fourth, the outcome variable — WHO full-vaccination coverage — reflects completion of the primary vaccination series as defined in each national context. Because the number of doses required for full vaccination differed across vaccine products and countries, cross-vaccine comparisons should be interpreted with additional caution.

Finally, the dataset captured the latest available coverage snapshot for each country rather than the trajectory of coverage over time. Countries that introduced vaccines earlier may have achieved higher cumulative coverage for reasons unrelated to vaccine type, and this temporal dimension was not modelled explicitly.

### 5.3 Implications

The findings of this study have two practical implications for the interpretation of global vaccination data. First, observed associations between vaccine type and national vaccination coverage should not be taken at face value as evidence that certain vaccine platforms are inherently more effective at achieving population-level uptake. Such associations are strongly confounded by the structural conditions under which different vaccines were procured and deployed, and analyses that do not adjust for these conditions risk attributing to vaccine category what is more appropriately attributed to national economic and logistical capacity.

Second, the results underscore the importance of equitable vaccine allocation in future pandemic responses. The near-complete confound between mRNA vaccine availability and national income suggests that the global allocation of vaccine platforms during the COVID-19 pandemic was closely tied to purchasing power rather than to population need or epidemiological criteria. Efforts to ensure broader access to diverse vaccine platforms across income settings may help reduce structural inequalities in future vaccination campaigns.

Future work could extend this analysis by incorporating additional country-level covariates, including health-system indicators, supply-chain measures, and vaccine confidence data, and by examining temporal patterns of coverage accumulation rather than a single endpoint snapshot. Individual-level or subnational analyses would be needed to assess whether any residual vaccine-type association persists after fuller adjustment for contextual factors.

## Supporting information

S1 Data WHO country–vaccine entries for manuscript. This file contains the cleaned country–vaccine-entry dataset used for the main manuscript analyses.
(CSV)

S2 Data WHO country–vaccine entries, latest snapshot. This file contains the latest country-level vaccination coverage records merged with vaccine-product introduction data.
(CSV)

S3 Data WHO country–vaccine entries monthly panel, 2021–2023. This file contains the expanded monthly country–vaccine-entry panel generated for reproducibility and sensitivity-check purposes.
(CSV)

S1 Code Python script for dataset construction, analyses, and figures. This file contains the Python code used to construct the analytical outputs and generate the figures used in the manuscript.
(PY)
